# Surgery

**Published:** 1904-03

**Authors:** J. Lacy Firth


### SURGERY.

**The cure of chronic Bright's disease by renal decapsulation.** Important papers dealing with this operation have been published since the subject was discussed in the pages of this *Journal* in December, 1902, especially another from the pen of G. M. Edebohls, the pioneer of the procedure. We shall give below a resumé of the most important papers by that writer on the subject, endeavouring on their basis to place before our readers the results of the operation and its indications and limitations in so far as these matters can be at present estimated. Later we shall state the views of some other surgeons on the subject.

It will be remembered that Edebohls, having found that nephropexy with decapsulation for movable kidney associated with chronic Bright's disease led to a cure of the nephritis, conceived the idea that the decapsulation, not the nephropexy, was mainly responsible for the good results obtained, and that decapsulation acted by removing a barrier to the creation of a new and increased blood supply to the diseased kidney. The removal of inflammatory products by absorption and the restoration of health to the kidney seemed to be the direct results of this increased blood supply and the improved circulation in the organ.

In December, 1901,[1] Edebohls reported in some detail nineteen cases in which he had decapsulated kidneys affected with chronic Bright's disease. In sixteen of these nephropexy was also performed. The list included eight cases in which a cure of Bright's disease lasting from one to over eight years after operation had been obtained. In March, 1903,[2] the same writer published the results he had obtained in thirty-two additional cases, bringing the total number to fifty-one. This included all the cases he had operated upon to the end of the year 1902.

[1] *Med. Rec.*, 1901, lx. 961. [2] *Ibid.*, 1903, lxiii. 481.

**Technique of the operation.** Without going fully into the details of the technique of the operation as recommended by Edebohls, the following points are worth special mention: The patient is placed prone, with a cylindrical air-cushion supporting the abdomen. By this arrangement both kidneys become accessible without changing the patient's position. If possible the kidney is lifted out of its fatty capsule bed and delivered through the wound. The capsule proper is then incised along the convex border of the kidney, and reflected from each side to the hilum and cut away. The kidney is then dropped back into its fatty capsule and the wound of the parietes closed, usually without drainage. Both kidneys are operated upon at the same time, and the operation should be completed in the maximum time of one hour. Difficulty may arise from the great length and obliquity of the twelfth rib; from the firm attachment of the kidney in its normal site, in which case it may be impossible to expose more than the lower pole; and from the firm attachment of the capsule to the kidney in the cases of the interstitial variety of nephritis. Great care must be taken not to tear away pieces of the kidney. The best instrument for reflecting the capsule is the index finger of the rubber-gloved hand. Nephropexy should only be performed if symptoms due to the mobility of the kidney call urgently for relief. Ether has proved to be a satisfactory anæsthetic. The congestive effect of the anæsthetic on the kidneys is counteracted by the moderate escape of blood from them during the operation.

**Condition of the patients operated upon, and the evidences of existing Bright's disease.** In Edebohls's first table of eighteen cases all the patients were women varying between nineteen and forty-five years of age. In sixteen of these nephropexy, either unilateral or bilateral, as well as decapsulation, was performed. The author classifies chronic Bright's disease as follows, viz.: (*a*) As interstitial nephritis those in which the gross evidences of inflammation of the connective tissue predominated; (*b*) As parenchymatous those in which involvement of the secretory apparatus predominated; and (*c*) As diffuse nephritis those in which the parenchyma and connective tissue of the organ were involved in a fairly equal degree. These varieties of inflammation are said to be easily recognisable during operation after a little experience. In the first eighteen cases alluded to, the chronic Bright's disease was found to be unilateral in eight. In nine of the eighteen cases Bright's disease was only discovered a short time before operation by examination of the urine. We may take it for granted that these were not severe cases. The other nine patients were fully aware that they had had Bright's disease for periods varying from several months to over six years, and they had dropsy and uræmia in varying degrees.

In 1902 Edebohls operated on thirty-two cases, in all of which the chronic Bright's disease was of an advanced type.

Nearly all presented cardiac and vascular degenerations of greater or less degree—arterio-sclerosis, hypertrophy of the heart of all degrees up to the point of non-compensation, dilatation, pericarditis and endocarditis. Some had pleuritis and hydrothorax, some hemiplegia, and some characteristic retinal lesions.

**Mortality of the operation.** Of the fifty-one patients fourteen died at periods of time after operation varying between twelve hours and eight years. Seven of these deaths occurred at periods varying from two months to eight years after operation. The other seven followed the operation at periods varying from twelve hours to fifteen days, and represent the operative mortality in the fifty-one cases. Edebohls therefore writes that his operative mortality may be stated as $13\frac{2}{3}$ per cent.; but in the first nineteen cases there was no mortality due to operation. In other words, the author lost seven cases out of thirty-two operated upon in 1902, which works out to a percentage of 21.87. Further, it may be noticed here that twelve patients, or 23.5 per cent., failed to survive the operation longer than one year and six days. The author points out that he was compelled to accept cases for operation in which a fatal result was almost a foregone conclusion. Some such cases, however, had made unexpected and surprisingly good recoveries. In fact, with two exceptions, the author had operated on every patient who had come to him desiring operation. One surgeon informed Edebohls that he had performed four decapsulations for chronic Bright's disease, and that each of the four had died promptly after the operation. These four operations represented exactly the half of the total experience of the surgeon in question in renal surgery.

**The therapeutic results of renal decapsulation.** Edebohls has been able to follow up the after-history in all but 3—*i.e.* in 48—of his 51 cases. Of these 48 cases, 24 at the time of writing—viz. March, 1903—could be considered available for estimating the final results of the operation. Of the 24 remaining, 3 had been lost sight of, 13 had died since operation, and 8 had only been operated on during the immediately preceding seven months, a period too short to give valuable data. Patients with chronic Bright's disease often improve temporarily, even for months at a time, either as a result of treatment or without treatment. The author considers, therefore, that to prove that cure or improvement is the result of renal decapsulation it must be shown: first, that cure or improvement follows the operation with practical uniformity; second, that a cure once obtained is, as a rule, lasting; third, that improvement attained by operation is steadily progressive in the great majority of cases. Judged by these standards, the author states that the therapeutic results have only been more or less unsatisfactory in 2 of the 24 patients available for the study of the question. One of these two cases, a patient who had had the disease under treatment for six years before operation lost all albumin in the urine a year after operation, and remained free from albuminuria and in apparently perfect health for nearly four years; then, in 1902, the albumin and casts reappeared. Only one of her kidneys had been operated upon. The other unsatisfactory case improved for eight months after operation; then she had a series of exacerbations in association with an attack of diphtheria, and with two or three attacks of grippe. Of the remaining 22 patients, 10 were radically cured and 12 were greatly improved. One of the cured patients died after an operation for tubal pregnancy twelve months after the kidney operation. Her urine was normal at the time of this second operation. Thus nine patients remain now living in good health, with urine free from albumin, at periods varying from one year and nine months to ten years after operation. The author is of opinion that the following conditions must be fulfilled in a given case in order that it may be pronounced cured: The urine must remain free from albumin and casts, and the daily urea output be normal, or approximately so, for a period of six months following the verification of the disappearance of albumin and casts; and the patient must be free from the symptoms of chronic Bright's disease from which he or she previously suffered. The above nine cases fulfilled these conditions. Unfortunately the nine cases belong to the earlier group of nineteen cases published by Edebohls in December, 1901, in which the disease was of a milder type and always associated with mobility of the kidney, and it seems open to question if they should be placed in the same category as ordinary bilateral chronic Bright's disease. In the 1902 series of cases, all were inveterate cases of complicated and far-advanced Bright's disease. The final results of the 1902 series of cases will therefore be of more value than those given above in deciding whether the operation is to be recommended for ordinary cases. The author says that, taking into consideration the severity of these cases, the sum total of improvement presented by the surviving patients of 1902 must be considered remarkably gratifying, and gives rise to the hope that a number of them may figure in the list of cures in the next report, for a number of them appear to be on the high road to perfect cure. When cases improve after the operation the first effect on the urine is shown by an increased daily out-put of urea. For example, a daily excretion of six grams before operation has been known to increase to one of thirty to thirty-five grams within a month. The improvement in the excretion occurs rapidly in the first two or three months, but continues steadily after that period. The epithelial, waxy, fatty and pus casts disappear gradually in from a month to a year. Albumin usually persists for a greater or less time after the permanent disappearance of all casts. The patient's general health improves *pari passu* with that of the urine.

**The selection of cases for operation.** The precise limits of the disease beyond which renal decapsulation cannot avail to save the patient's life, cannot as yet be positively stated. In several cases the operation has proved a boon to patients by diminishing cardiac hypertrophy. But if cardiac and vascular changes have advanced too far ever to be made good or even improved, the operation becomes so dangerous that it is not to be advised. The mortality attending the operation will be that of the disease itself and its complications, for the operation is almost without danger when properly performed. Speaking in May, 1903,[1] Edebohls said that in his published articles he had tried to subdue his enthusiasm, but his results had been better than pictured; all patients had been relieved of their headaches and backaches, and the circulation and heart's action had been improved. He had had an opportunity of making serial sections of the kidneys operated upon four months previously. Enormously dilated blood vessels were shown penetrating the kidney from the fatty capsule.

**Unilateral chronic nephritis.** In his paper of December, 1901, Edebohls stated that the chronic nephritis affected both kidneys in nine cases and only one kidney in eight cases. The evidence of the disease being unilateral in six of the eight cases was derived from an examination of both organs at the operation by sight and touch. In the other two the patients recovered completely, although only the right kidney was operated upon. This evidence we consider weak. The author believes that this one-sidedness of the disease explains the chronic and mild course of some cases of Bright's disease. The healthy kidney continues to perform the eliminative work of both organs. The disease probably invariably becomes bilateral in the course of time. When a fatal result ensues the *post-mortem* examinations will practically always show bilateral disease. Hence the scepticism of physicians as to the existence of unilateral chronic Bright's disease. It will be interesting, in conclusion, to glance at some of the published views of other surgeons on the value of the procedures under consideration.

Ramón Guiteras (New York)[2] began to perform these operations in 1902 as a sceptic, and had refused to operate on many cases which he would now consider deserving of operation. He had sent a circular letter to ascertain the views of leading surgeons on the question. From the 150 answers received it appeared that those in favour of the operation and those against it were about equally divided. Over 100 cases of the operation had been reported—16 per cent. as cured, 40 per cent as improved, 11 per cent. as unimproved, and 33 per cent. as fatal. Many cases of albuminuria and cylindruria were not, properly speaking, Bright's disease. Movable kidney was often associated with this condition, and nephropexy might then absolutely cure it. It was generally recognised by pathologists that true

[1] *Med. Rec.*, 1903, lxiv. 475. [2] *Med. Rec.*, 1903, lxiv. 474.

Bright's disease was always bilateral. In some cases he had found evidence of nephritis of both kidneys by ureteral catheterisation and yet nephropexy on one side led to the common urine from both organs becoming free from the signs of Bright's disease, except that the percentage of urea did not come up to the normal. This might be explained by a sympathy between the two kidneys, like that between the two eyes (Pousson). He was not sure whether it was better to partially decapsulate and fix the kidneys or to entirely decapsulate and return them into the fatty capsule. According to Johnson, after decapsulation of the kidneys in dogs, a fibrous investment was reformed for the kidneys which macroscopically resembled the normal capsule. In cases of anasarca with a bad heart it was a question whether one should operate or not. The results in such cases showed that 50 per cent. died, 25 per cent. were unimproved, and 25 per cent. were cured or improved. He had never had the courage to operate in such cases.

A. H. Ferguson (Chicago)[1] reports sixteen cases of decapsulation, mostly combined with nephropexy, and that on one side only, for movable kidney and nephritis. The type of the disease was mild in these cases. He writes: "The results so far have been excellent and astonishingly prompt."

H. Kümmel and O. Rumpel (Hamburg)[2] regard it as extremely difficult during an operation on a kidney to diagnose its condition, unless striking naked-eye changes are visible, as in some examples of granular kidney. Those authors have investigated thirty-five cases of nephritis with the aid of cryoscopy, and twelve of these with the additional aid of the ureteral catheter. Nephritis, whether parenchymatous or interstitial, they have always found to be bilateral. They report three cases of decapsulation for the disease. One case, in which they decapsulated the left kidney only, derived no benefit, and died five months later of uræmia. The other two cases seemed to be benefited, though one was not observed long enough to be of any value in judging of the final results.

Jaboulay[3] relates a striking case of improvement after unilateral decapsulation. There had been albuminuria for a year or fifteen months before the operation; after that event it ceased in twelve days. The writer advances the hypothesis that these operations act by the traumatism they inflict on the sympathetic system situated in the renal pedicle. The rapidity of the improvement in the reported case is considered to be best explained by this theory.

F. G. Balch (Boston)[4] reports nine cases. The disease was of the chronic diffuse type. One case was probably cured, though it is too early to be sure of this. One was improving

[1] *J. Am. M. Ass.*, 1903, xli. 8.
[2] *Beitr. z. klin. Chir.*, Band xxxvii., Heft iii. 968.
[3] *Arch. gén. de Méd.*, November 17th, 1903; vide *Brit. M. J.*, 1904, i., Epitome, p. 10.
[4] *Boston M. and S. J.*, 1904, cl. 90.

eleven months after operation. Two cases a year after the operation showed little if any improvement. The other five died at varying periods after the operation, viz., one each at fourteen months, five days, three months, two months, and one at a period not clearly stated, perhaps three months. Thus the results in these nine cases, only two being improved, were not of a very gratifying kind.

Joshua C. Hubbard (Boston)[1] reports seven cases. One of these may be rejected, as the patient died of tuberculosis of the lungs and kidneys six months later. Of the six remaining cases four died two and nineteen days, and one and three months respectively after operation. In the remaining two cases the results were satisfactory, for in one, although there was a trace of albumin a year and four months after, there were no casts in the urine and the patient was free from symptoms; in the other the patient at the end of seven months had $\frac{1}{4}$ per cent. albumin, but felt well and was engaged in arduous work.

J. LACY FIRTH.

---

